# Supplementary material for: High-throughput phenotyping methods for quantifying hair fiber morphology
Source: Sci Rep. 2021 Jun 1;11:11535. doi: 10.1038/s41598-021-90409-x (PMC8169905; doi:10.1038/s41598-021-90409-x)
Supplement: Supplementary file 2 — Supplementary Information 2. [file 41598_2021_90409_MOESM2_ESM.pdf]

High-throughput phenotyping methods for quantifying hair fiber morphology

Tina Lasisi[1]*, Arslan A. Zaidi[2], Timothy H. Webster[3], Nicholas B. Stephens[1], Kendall Routch[1], Nina G. Jablonski[1], Mark D. Shriver[1]

[1] Department of Anthropology, Pennsylvania State University
[2] Department of Genetics, Perelman School of Medicine, University of Pennsylvania
[3] Department of Anthropology, University of Utah
* Tina Lasisi: tpl5158@psu.edu

Supplementary Results 2

Biological Significance

Tina Lasisi

2021-05-06 10:38:38

To explore the significance of quantifying hair fiber morphology, we explore the relationship between various quantitative hair traits, categorical data and genotype data on the same sample.

Our data consists of 193 individuals for whom we have quantitative hair phenotype data. In our first data quality control step, we filter to keep individuals who have more than 4 hair fragments in their curvature image and over 10% African ancestry. We calculate mean and median values for the cross-sectional data we have collected for individuals (~ 6 sectioned hair fibers). In our analyses, we use median values as they are less affected by intra-individual outliers.

Self-reported hair texture vs. quantitative hair curvature

We compare the self-reported hair texture with mean and median curvature for our sample.

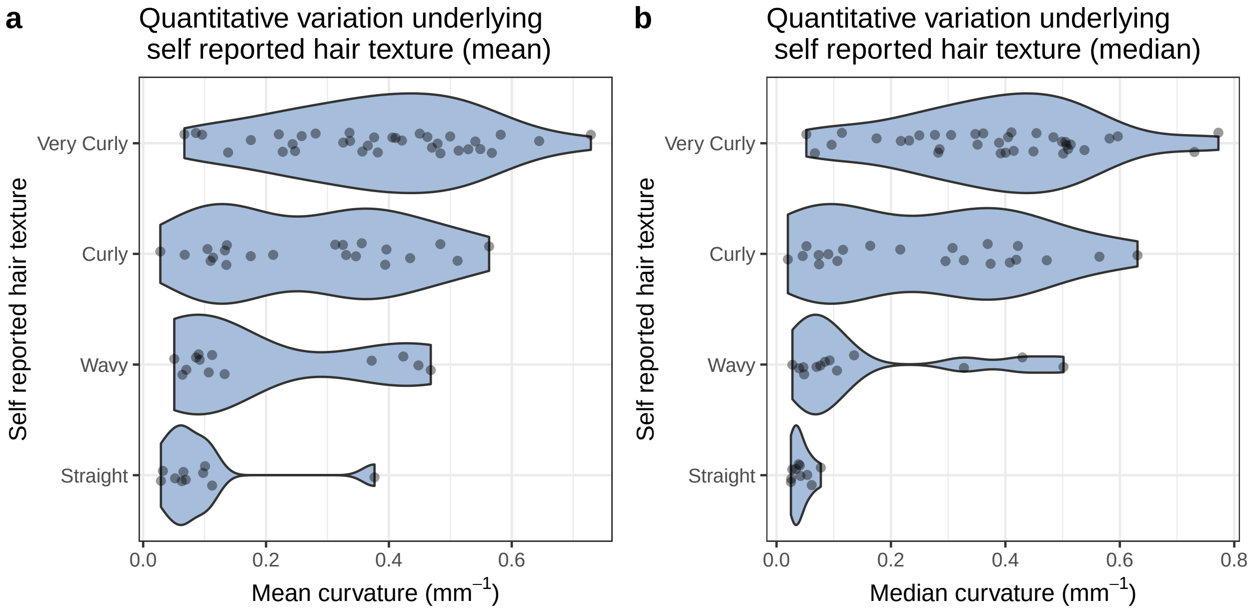

Figure 1: Self-reported hair texture vs. quantitative hair curvature

The single individual with a high mean curvature in the straight group is the result of an artefact in the image.

The red arrow points to a stray fiber that likely contaminated the sample and was missed during imaging. Such potential outliers are the reason we chose to use the median curvature for a sample in our analyses.

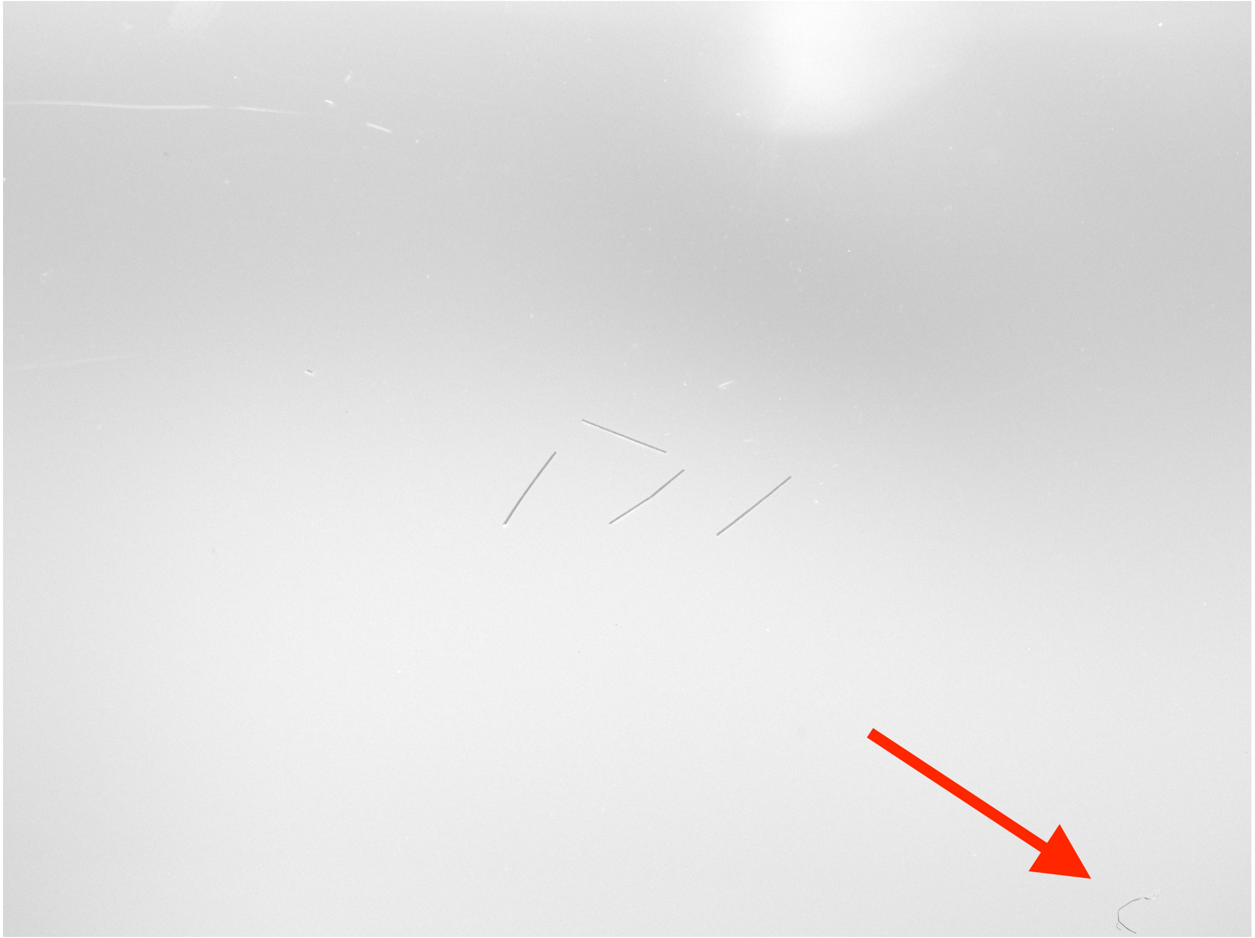

Figure 2: Image of hair sample with artefact biasing the measurement

Objective hair texture classification vs. quantitative curvature

To explore how much data is lost when binning continuous variation, we compared mean and median curvature to classified hair texture. This classification is based on Loussouarn et al.'s 2007 paper "Worldwide diversity of hair curliness: a new method of assessment.

While the authors propose a number of parameters to distinguish curlier hair types (based on number of twists and waves among other factors), their primary classification is based on curvature. We demonstrate that, regardless of additional parameters, a considerable range of curvature is obscured when collapsing hair variation according to their curvature thresholds.

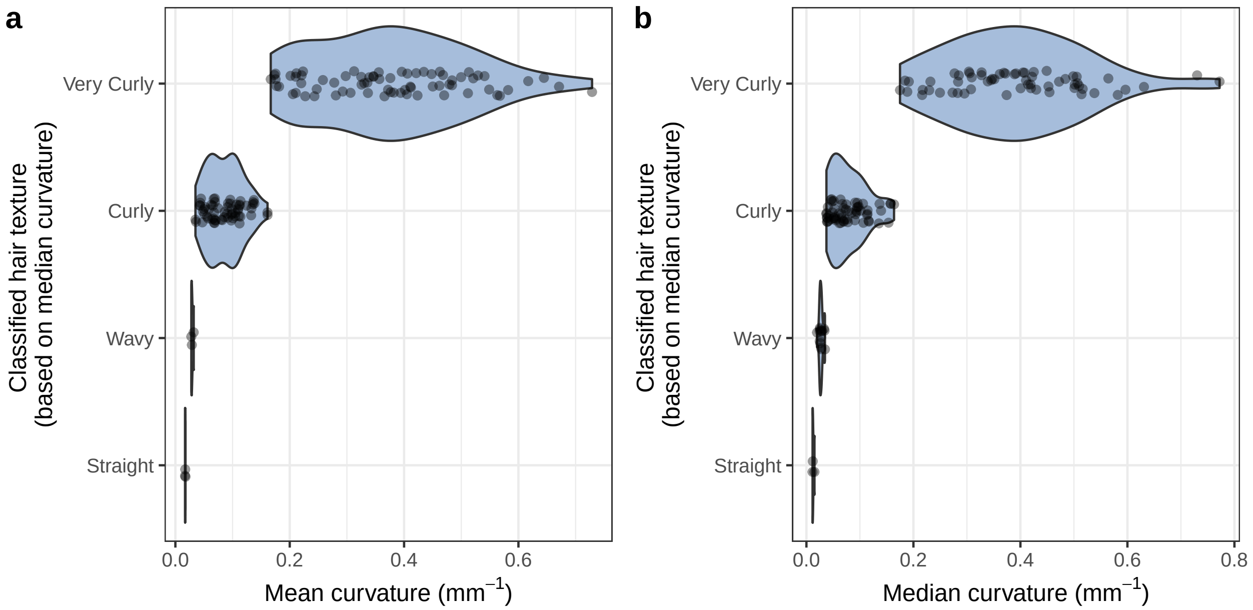

Figure 3: Objective hair classification vs. quantitative curvature

Ancestry vs. hair morphology

We carried out a number of analyses using the genotype data collected for this diverse sample. In an admixed sample where a continuous trait has divergent distributions in the parental ancestry groups, the resulting admixed population can show a correlation between ancestry and that trait. Finding such a correlation suggests may imply a polygenic trait with high heritability.

Admixture components

Our sample consists of admixed individuals with primarily African and European ancestry.

The colors represent ancestries that correspond to the following 1000 Genomes populations: - SAS = South Asian - AMR = American - AFR = African - EUR = European - EAS = East Asian

Each of these are metapopulations based on the grouping of multiple (sub)continental population groups in the 1000 Genomes repository.

Ancestry vs. curvature

Here we plot the correlation between proportion of African ancestry and m-index, median curvature, and eccentricity.

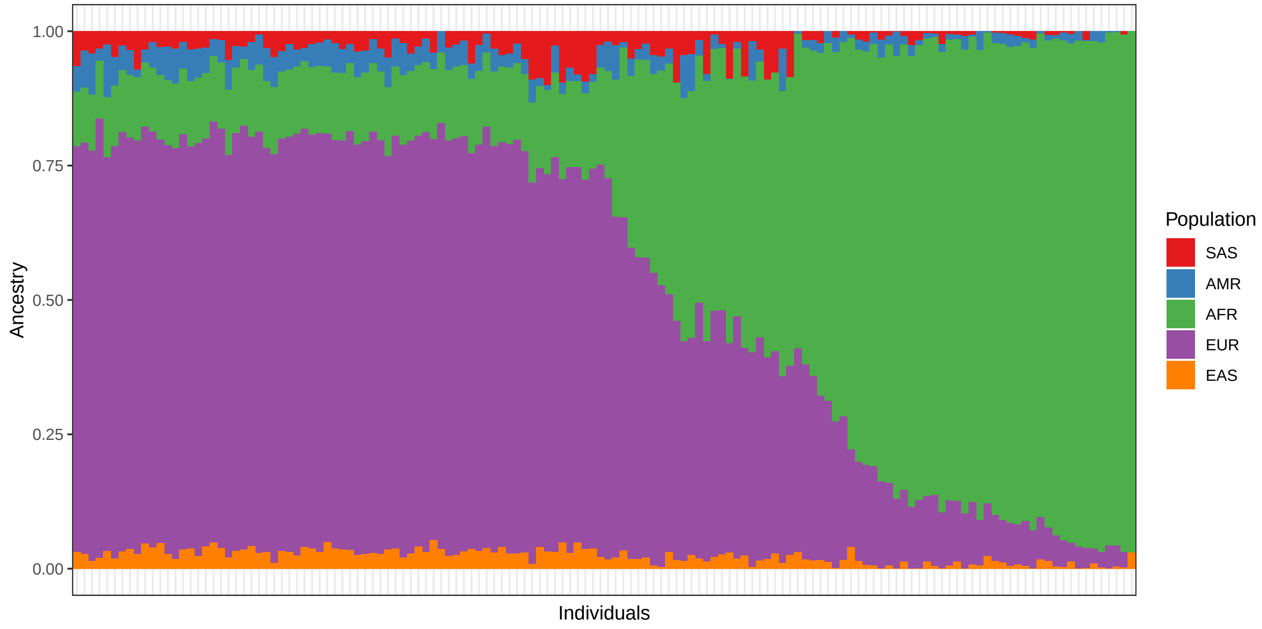

Figure 4: Admixture components for sample

Curvature vs. eccentricity

The relationship between cross-sectional shape (eccentricity) and curvature has long been debated. Due to the coincidence of cross-sectional shape and curvature in various populations that are often contrasted (i.e. East Asian vs. North European vs. West African), it has been unclear whether these traits have a causal relationship (specifically that higher eccentricity predicts higher curvature). In our admixed sample, we have the opportunity to test this question and fit a model between these traits with and without ancestry.

Uncorrected

First we examine the data without correcting for ancestry.

Fit a simple linear model and check the residuals.

```
##
## Call:
## lm(formula = curv_median ~ eccentricity_median, data = df_curv_ecc)
##
## Residuals:
##     Min      1Q  Median      3Q     Max
## -0.2843 -0.1245 -0.0264  0.1125  0.4770
##
## Coefficients:
##                       Estimate Std. Error t value Pr(>|t|)
## (Intercept)            -0.5682     0.1270  -4.474 1.94e-05 ***
## eccentricity_median     1.0284     0.1692   6.076 1.96e-08 ***
## ---
## Signif. codes:  0 '***' 0.001 '**' 0.01 '*' 0.05 '.' 0.1 ' ' 1
##
## Residual standard error: 0.1576 on 106 degrees of freedom
## Multiple R-squared:  0.2583, Adjusted R-squared:  0.2513
## F-statistic: 36.92 on 1 and 106 DF,  p-value: 1.959e-08
```

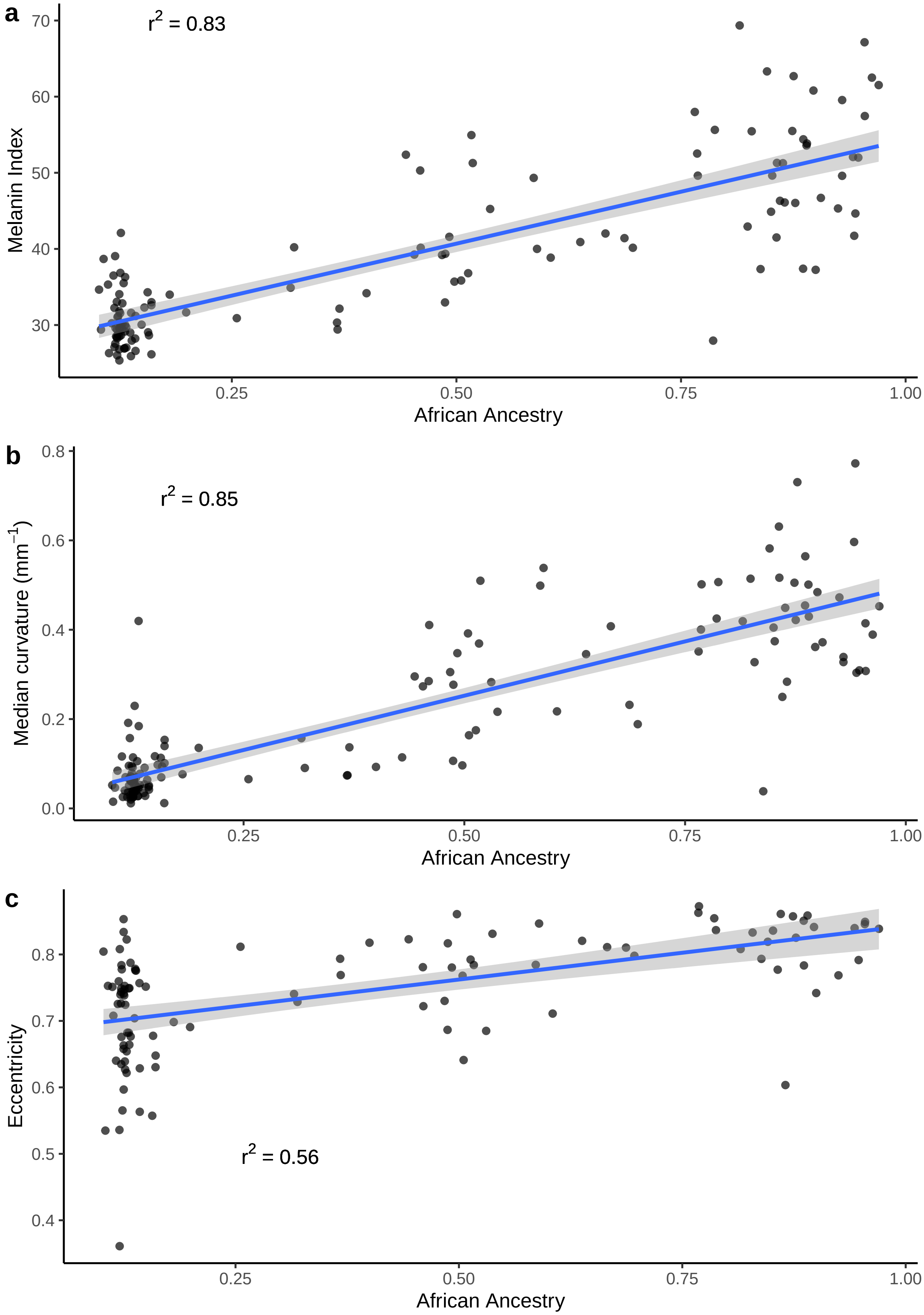

Figure 5: Percentage of African ancestry vs. M-index, curvature and eccentricity

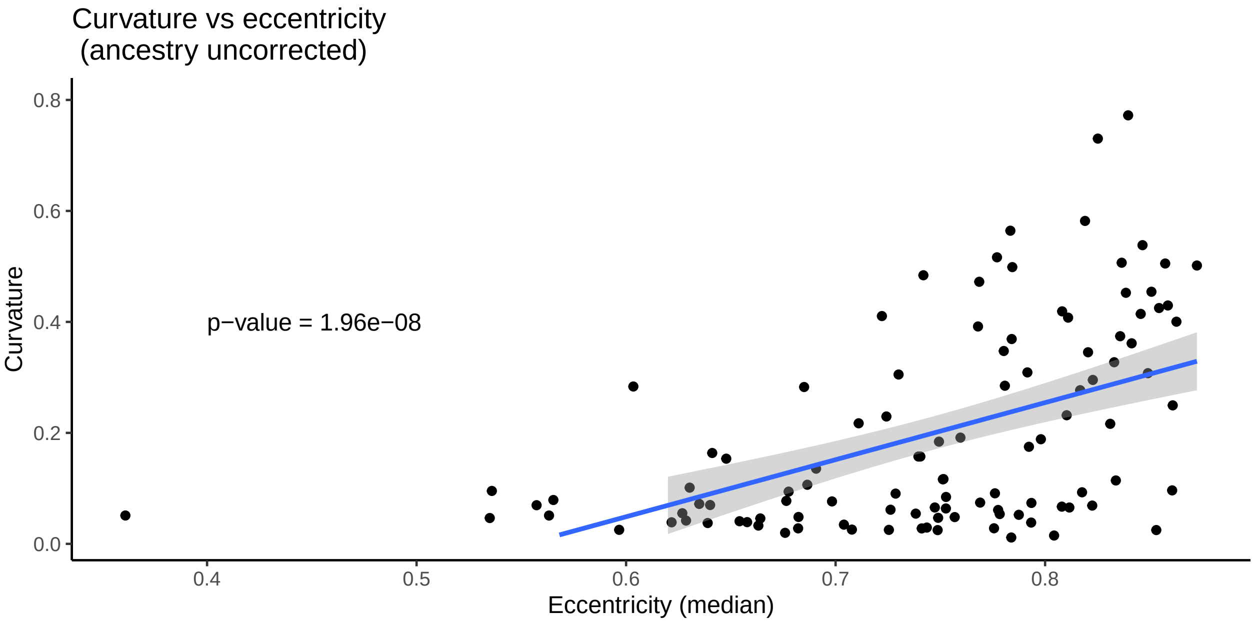

Figure 6: Curvature vs. eccentricity (without correction for ancestry)

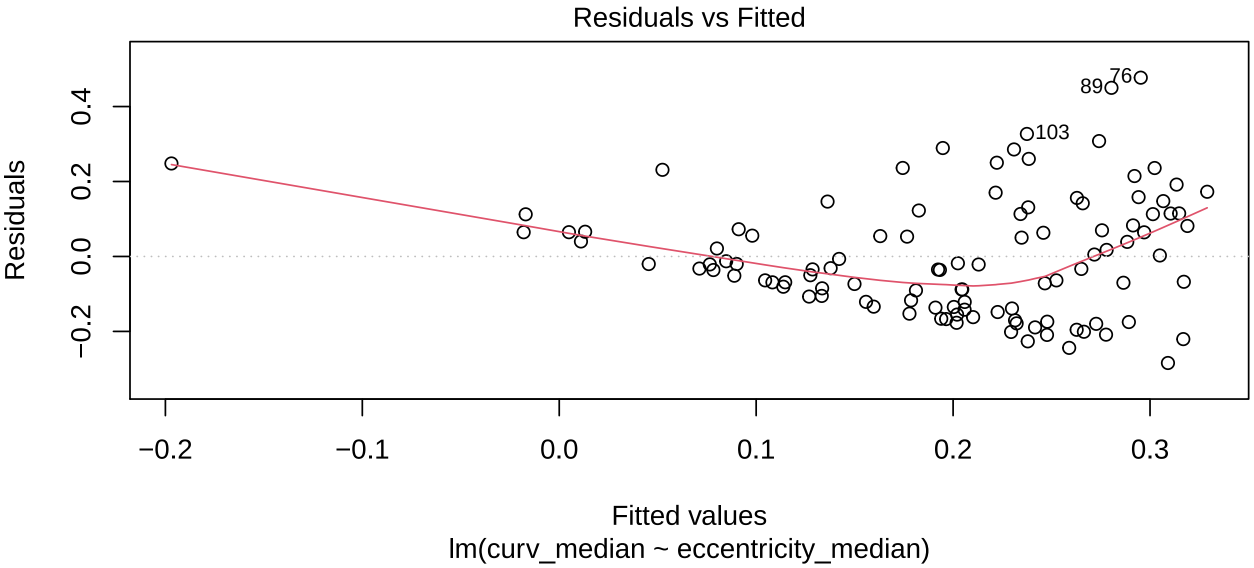

Figure 7: Residuals vs. fitted (linear model)

The residual plots show that there might be a few problems with the relationship: 1. possibly an outlier distorting the relationship, 2. relationship might not be linear 3. errors might be heteroescedastic.

We refit the model after removing the outlier.

```
##
## Call:
## lm(formula = curv_median ~ eccentricity_median, data = df_curv_ecc)
##
## Residuals:
##      Min       1Q   Median       3Q      Max
## -0.29590 -0.12290 -0.01791  0.09892  0.46713
##
## Coefficients:
##                     Estimate Std. Error t value Pr(>|t|)
## (Intercept)          -0.6707     0.1387  -4.836 4.56e-06 ***
## eccentricity_median   1.1622     0.1842   6.311 6.73e-09 ***
## ---
## Signif. codes:  0 '***' 0.001 '**' 0.01 '*' 0.05 '.' 0.1 ' ' 1
##
## Residual standard error: 0.1561 on 105 degrees of freedom
## Multiple R-squared:  0.275,  Adjusted R-squared:  0.2681
## F-statistic: 39.83 on 1 and 105 DF,  p-value: 6.731e-09
```

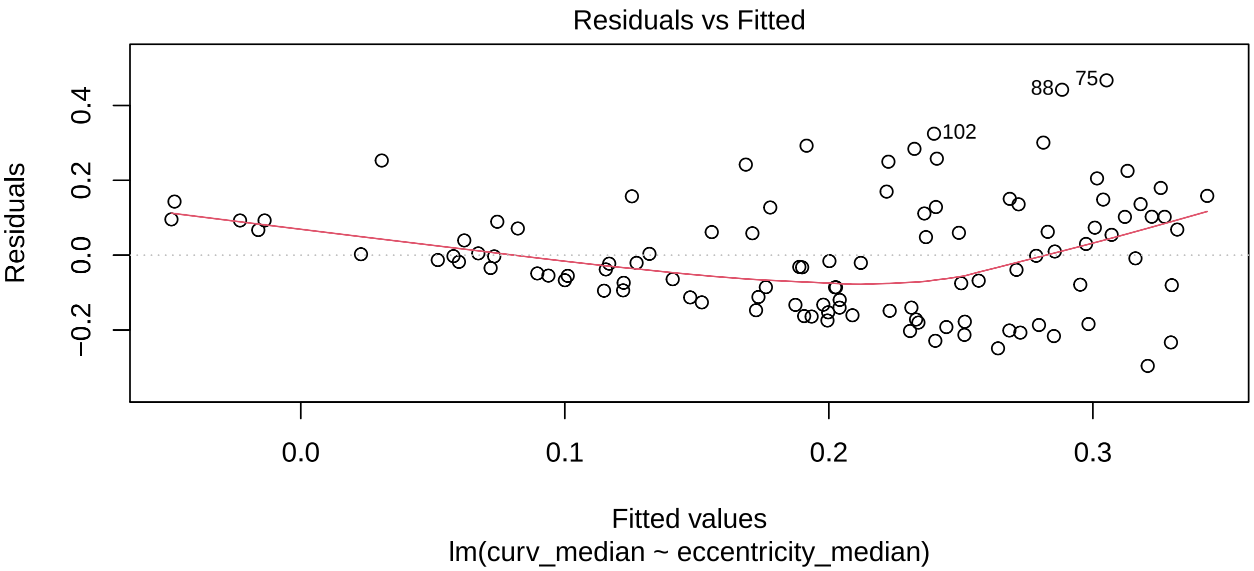

Figure 8: Residuals vs. fitted model

The outlier removal improves the fit. Next, check if the data fits a curvilinear (quadratic) model better.

The quadratic fit is better that the simple linear fit. Let's compare the two models formally and see if the quadratic fit is better.

```
## Analysis of Variance Table
##
## Model 1: curv_median ~ eccentricity_median
## Model 2: curv_median ~ eccentricity_median.c + eccentricity_median.c2
##   Res.Df    RSS Df Sum of Sq Pr(>Chi)
## 1    105 2.5589
```

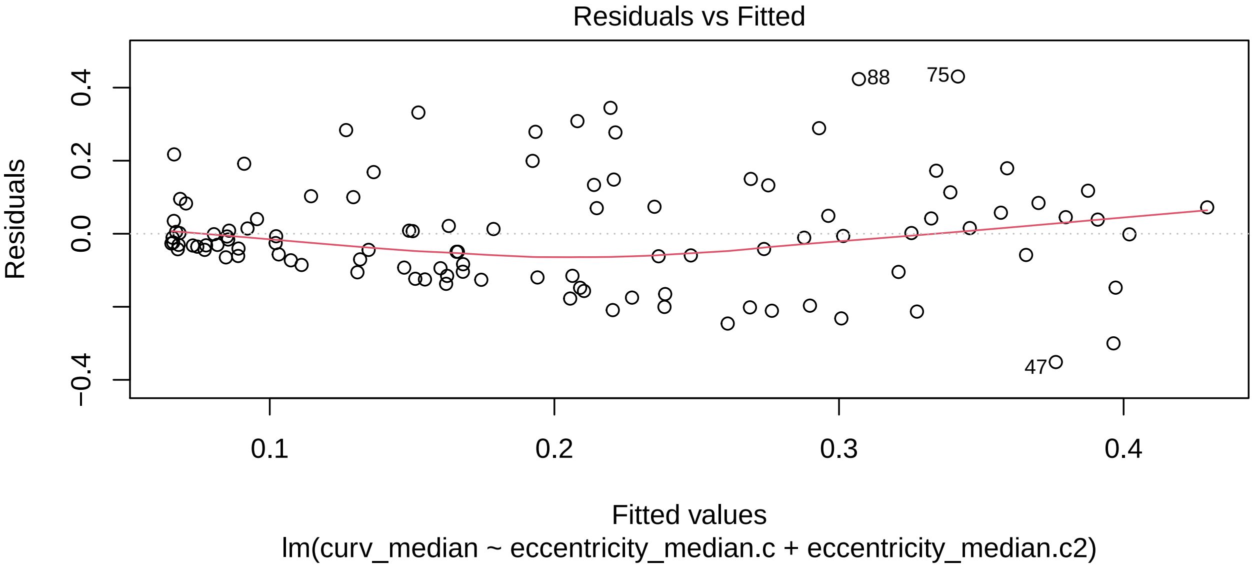

Figure 9: Residuals vs. fitted model (outlier removed)

```
## 2    104 2.3655  1   0.19343 0.003543 **
## ---
## Signif. codes:  0 '***' 0.001 '**' 0.01 '*' 0.05 '.' 0.1 ' ' 1
```

This shows that the residual sum of squares is significantly smaller for the quadratic fit, i.e., the quadratic model fits the data better.

There still seems to be some heteroescedasticity in the errors. We will use the fitted values as weights in a weighted regression. Because the variance increases with the fitted values, we will use $\frac{1}{fitted\ values^2}$ as weights.

```
##
## Call:
## lm(formula = curv_median ~ eccentricity_median.c + eccentricity_median.c2,
##     data = df_curv_ecc, weights = inv.curv)
##
## Weighted Residuals:
##     Min      1Q  Median      3Q     Max
## -0.9321 -0.6342 -0.1648  0.3443  3.1744
##
## Coefficients:
##                        Estimate Std. Error t value Pr(>|t|)
## (Intercept)             0.16298    0.01489  10.946  < 2e-16 ***
## eccentricity_median.c   1.35687    0.24357   5.571    2e-07 ***
## eccentricity_median.c2  5.06864    1.56175   3.245  0.00158 **
## ---
## Signif. codes:  0 '***' 0.001 '**' 0.01 '*' 0.05 '.' 0.1 ' ' 1
##
## Residual standard error: 0.8123 on 104 degrees of freedom
## Multiple R-squared:  0.2557, Adjusted R-squared:  0.2414
## F-statistic: 17.87 on 2 and 104 DF,  p-value: 2.14e-07
```

The variance in the residuals is more homogeneous under this model. We will re-generate the scatterplot with this model fit.

Thus, there is a significant relationship between eccentricity and curvature. Does this hold when we add

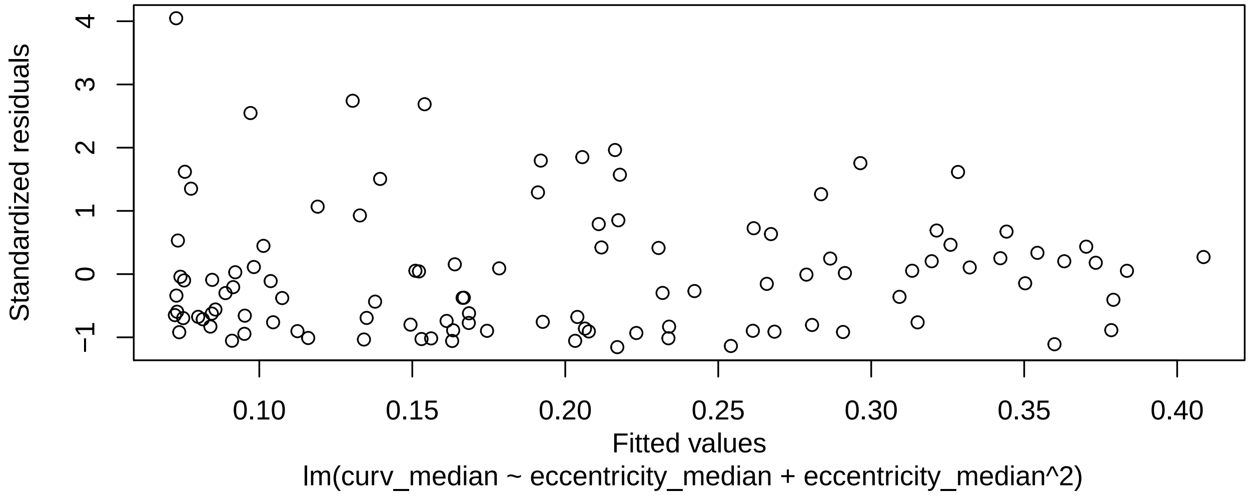

Figure 10: Residuals vs. fitted model (weighted regression)

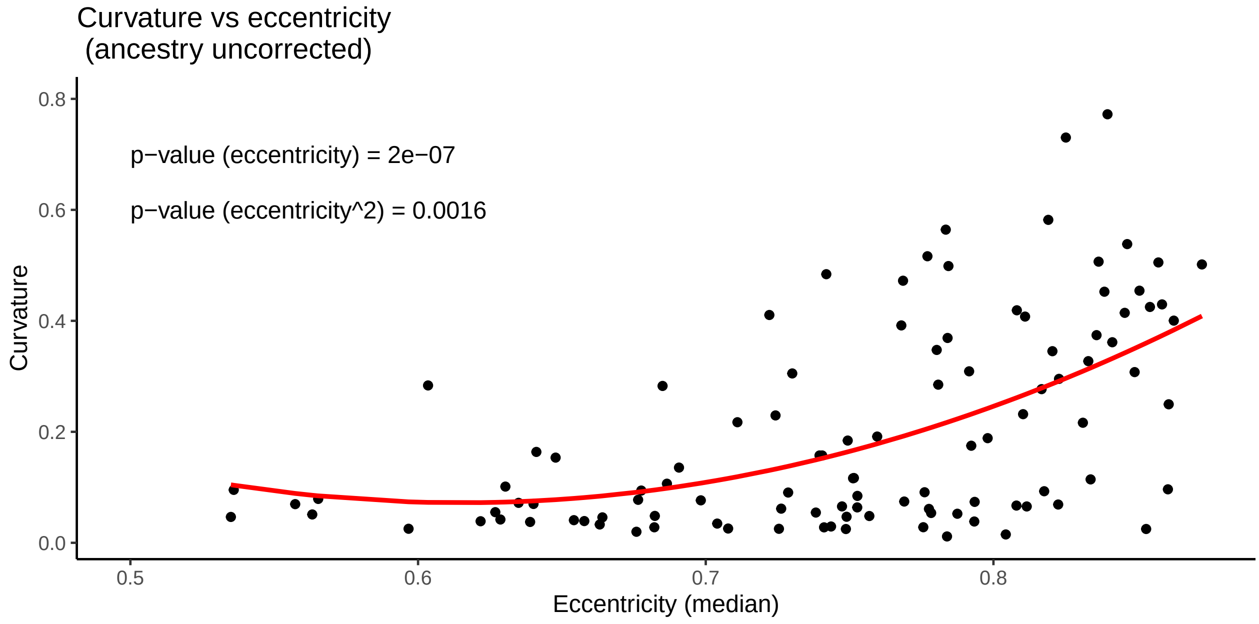

Figure 11: Curvature vs eccentricity (ancestry uncorrected) fit comparison

ancestry as a covariate in the model?

Corrected

We re-plot the relationship after residualizing curvature and eccentriciy (both) on ancestry.

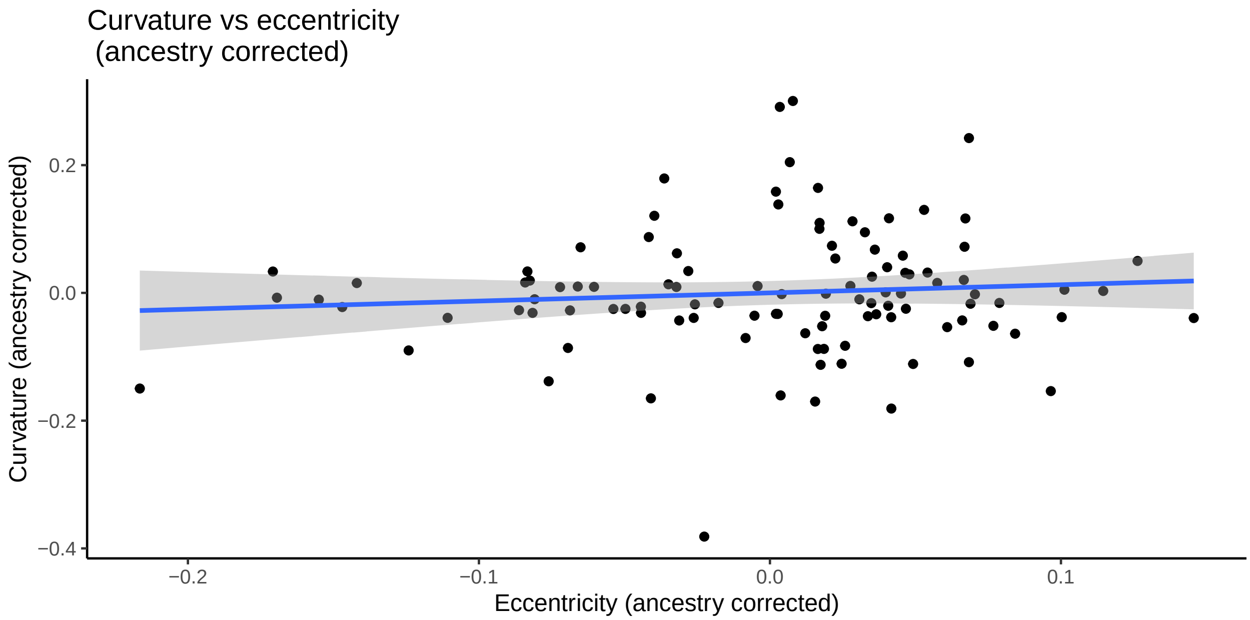

Figure 12: Curvature vs. eccentricity (with correction for ancestry)

When we correct for ancestry, the association between eccentricity and curvature is no longer significant. This supports the idea that these traits co-occur due to ancestry stratification. Let's test this formally.

The relationship looks rather linear so we will start with a simple linear model and inspect the residuals.

```
##
## Call:
## lm(formula = curv_median ~ eccentricity_median + AFR, data = df_curv_ecc)
##
## Residuals:
##      Min       1Q   Median       3Q      Max
## -0.37867 -0.04211 -0.00799  0.03639  0.29942
##
## Coefficients:
##                     Estimate Std. Error t value Pr(>|t|)
## (Intercept)         -0.08587    0.09768  -0.879    0.381
## eccentricity_median  0.12775    0.14012   0.912    0.364
## AFR                  0.47886    0.03716  12.885   <2e-16 ***
## ---
## Signif. codes:  0 '***' 0.001 '**' 0.01 '*' 0.05 '.' 0.1 ' ' 1
##
## Residual standard error: 0.09735 on 104 degrees of freedom
## Multiple R-squared:  0.7208, Adjusted R-squared:  0.7154
## F-statistic: 134.2 on 2 and 104 DF,  p-value: < 2.2e-16
```

The relationship looks pretty linear but there's still some heteroescedasticity in the residuals. We will again fit a weighted regression model.

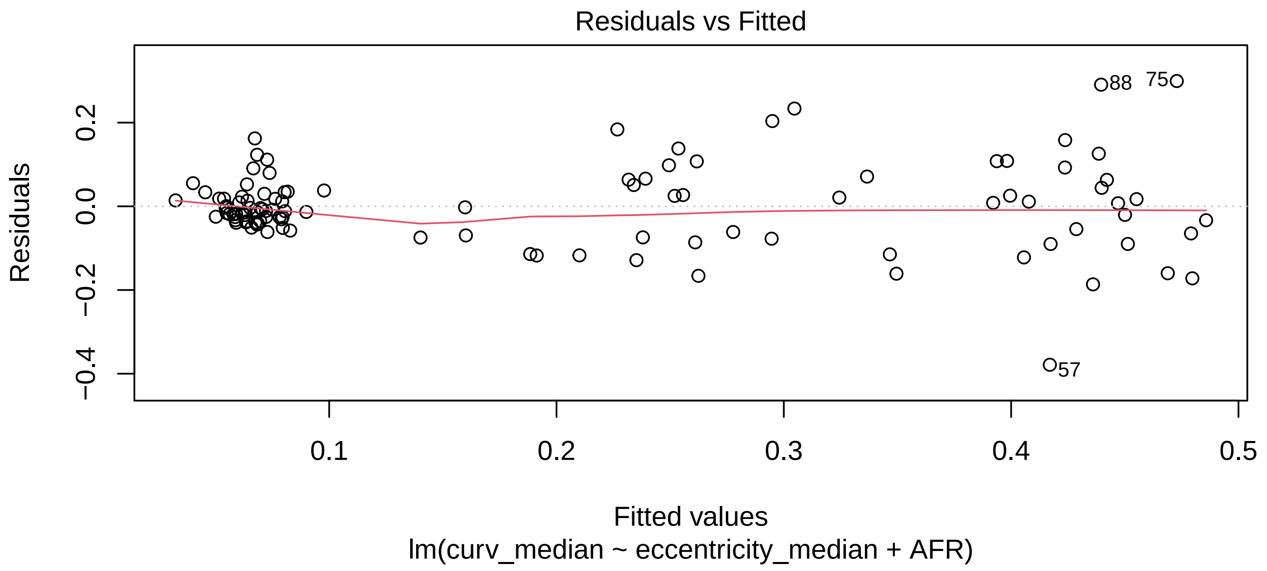

Figure 13: Curvature vs. eccentricity (with correction for ancestry)

```
##
## Call:
## lm(formula = curv_median ~ eccentricity_median + AFR, data = df_curv_ecc,
##     weights = fitted.cve_afr)
##
## Weighted Residuals:
##      Min       1Q   Median       3Q      Max
## -0.90751 -0.40872 -0.06239  0.25190  2.43315
##
## Coefficients:
##                     Estimate Std. Error t value Pr(>|t|)
## (Intercept)         -0.02120    0.03712  -0.571    0.569
## eccentricity_median  0.03448    0.05568   0.619    0.537
## AFR                  0.48972    0.05118   9.570 6.17e-16 ***
## ---
## Signif. codes:  0 '***' 0.001 '**' 0.01 '*' 0.05 '.' 0.1 ' ' 1
##
## Residual standard error: 0.5622 on 104 degrees of freedom
## Multiple R-squared:  0.4921, Adjusted R-squared:  0.4824
## F-statistic: 50.39 on 2 and 104 DF,  p-value: 5.003e-16
```

The weighted regression accounts for the heteroescedasticity.

Curvature vs. skin pigmentation

To demonstrate the potential effect of population stratification on traits, we compare hair curvature with skin pigmentation (m-index). These two traits are not biologically related, yet, in an admixed population, we may see a correlation that is due to population stratification of these polygenic traits.

Uncorrected

First we examine the relationship between curvature and skin pigmentation without correcting for ancestry.

```
##
```

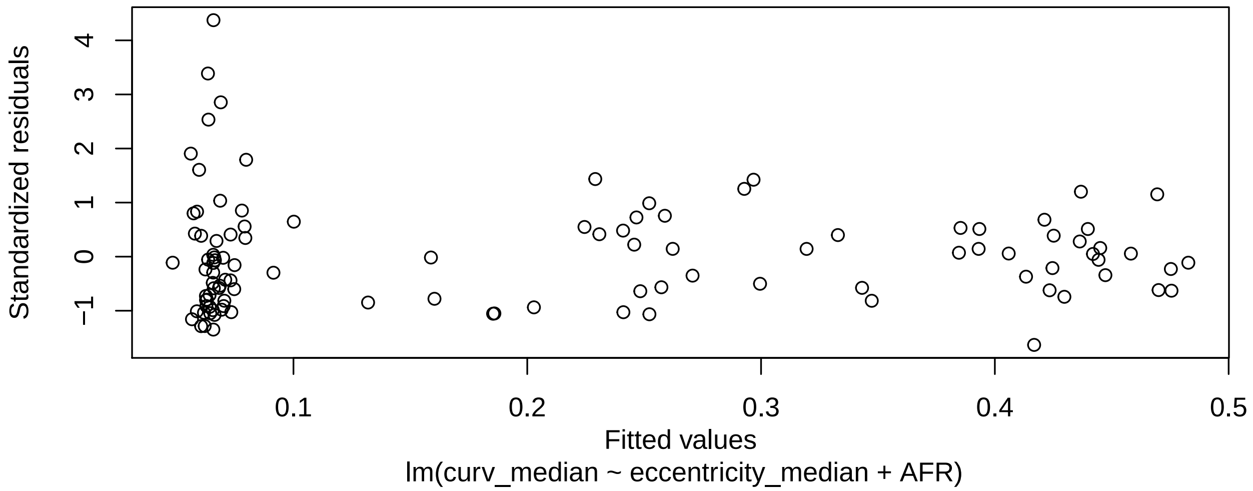

Figure 14: Residuals vs fitted values (Weighted regression model)

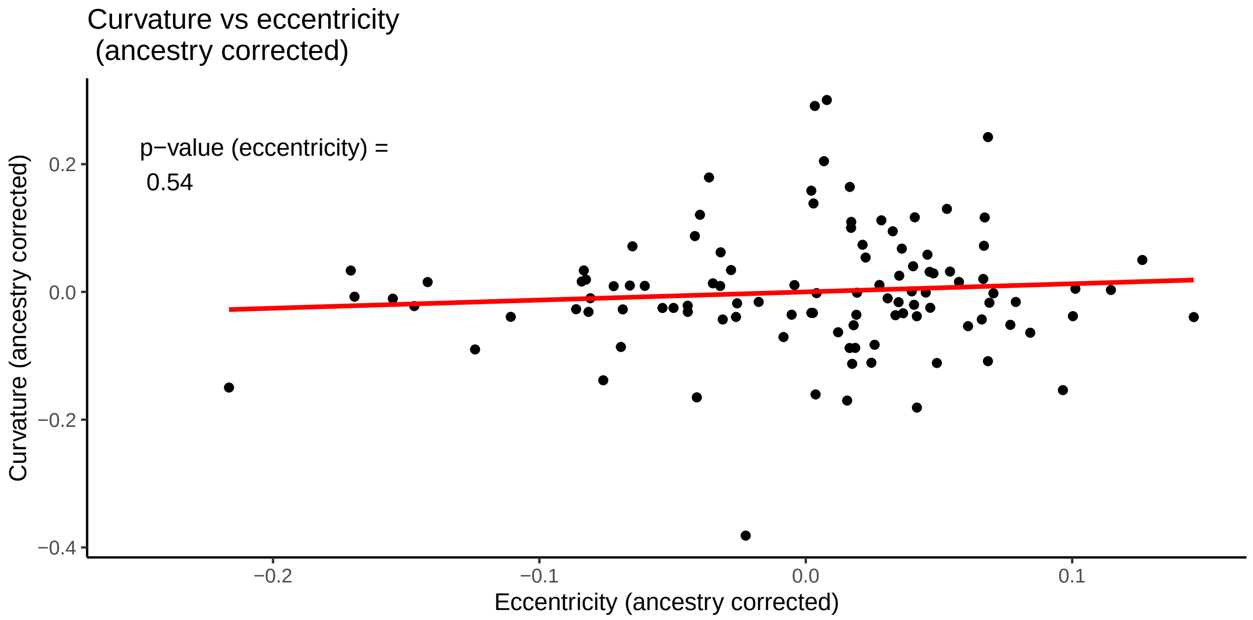

Figure 15: Curvature vs eccentricity (ancestry corrected)

```
## Call:
## lm(formula = curv_median ~ m_index, data = df_curv_mindex)
##
## Residuals:
##      Min       1Q   Median       3Q      Max
## -0.20715 -0.06804 -0.03471  0.04818  0.51483
##
## Coefficients:
##              Estimate Std. Error t value Pr(>|t|)
## (Intercept) -0.280232   0.041533  -6.747 4.91e-10 ***
## m_index      0.012887   0.001031  12.501  < 2e-16 ***
## ---
## Signif. codes:  0 '***' 0.001 '**' 0.01 '*' 0.05 '.' 0.1 ' ' 1
##
## Residual standard error: 0.1261 on 126 degrees of freedom
## Multiple R-squared:  0.5536, Adjusted R-squared:  0.5501
## F-statistic: 156.3 on 1 and 126 DF,  p-value: < 2.2e-16
```

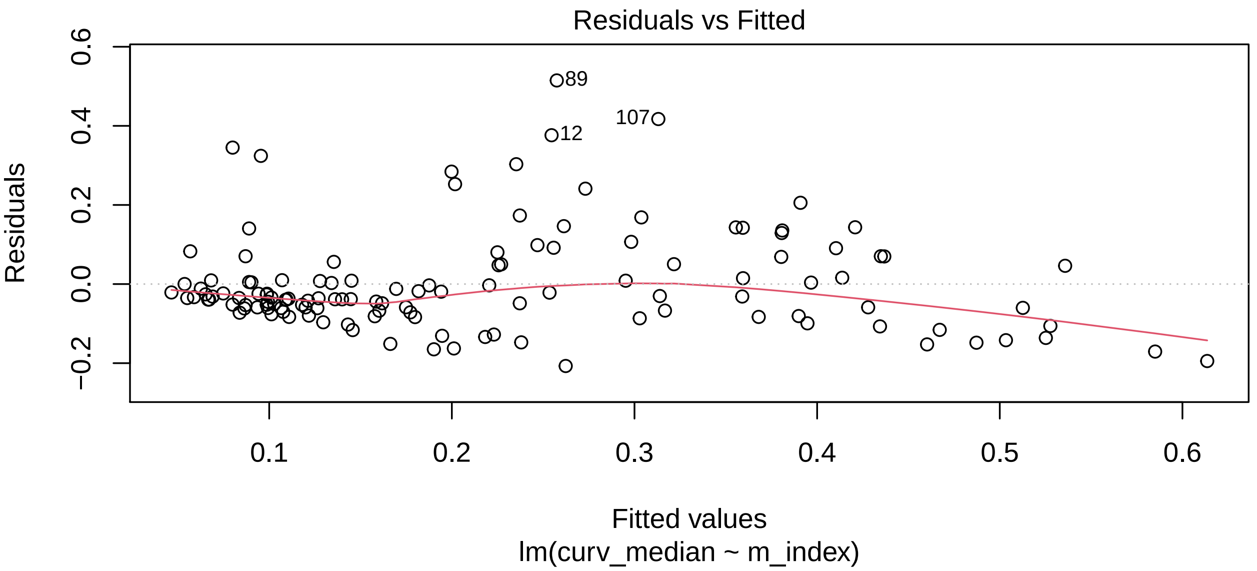

Figure 16: Residuals vs. fitted for M-Index and curvature (linear model)

As expected, we see a significant correlation between the two traits. The relationship appears to be linear but there seems to be more variance for observations with higher Melanin Index/curvature. We will use a weighted regression approach again.

```
##
## Call:
## lm(formula = curv_median ~ m_index, data = df_curv_mindex, weights = weights_cvm)
##
## Weighted Residuals:
##     Min      1Q  Median      3Q     Max
## -0.8842 -0.3855 -0.2268  0.1617  4.4355
##
## Coefficients:
##              Estimate Std. Error t value Pr(>|t|)
## (Intercept) -0.312749   0.047890  -6.531 1.46e-09 ***
## m_index      0.013704   0.001582   8.660 1.92e-14 ***
```

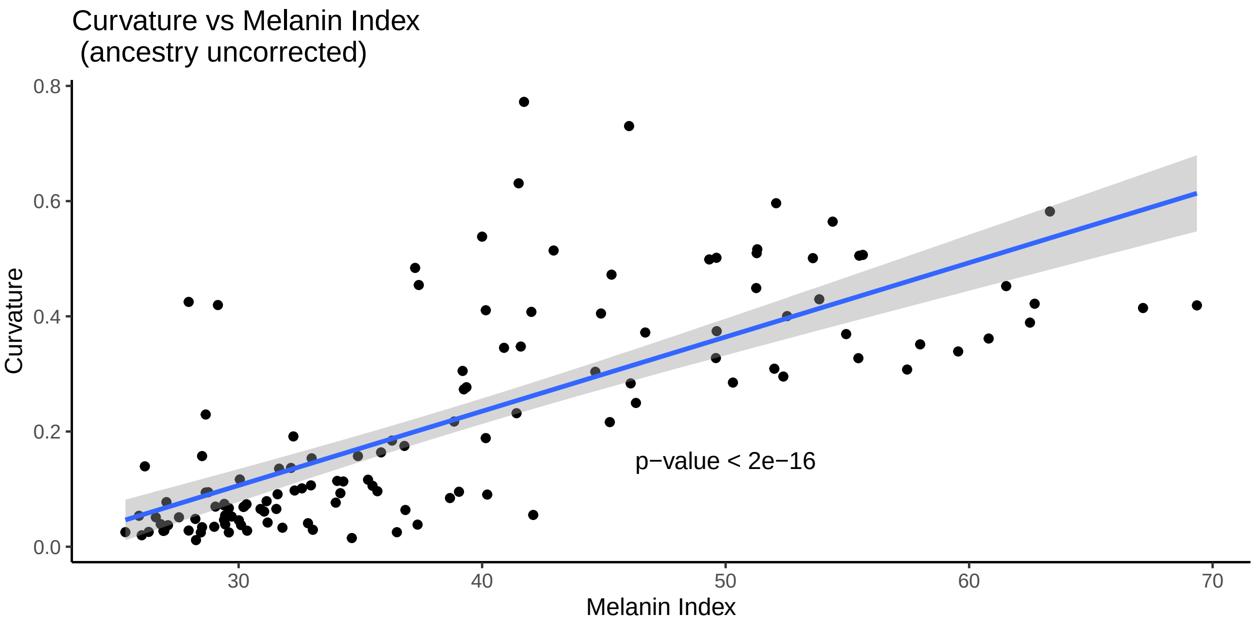

Figure 17: Curvature vs. M-index (without correction for ancestry)

```
## ---
## Signif. codes:  0 '***' 0.001 '**' 0.01 '*' 0.05 '.' 0.1 ' ' 1
##
## Residual standard error: 0.7457 on 126 degrees of freedom
## Multiple R-squared:  0.3731, Adjusted R-squared:  0.3681
## F-statistic: 74.99 on 1 and 126 DF,  p-value: 1.917e-14
```

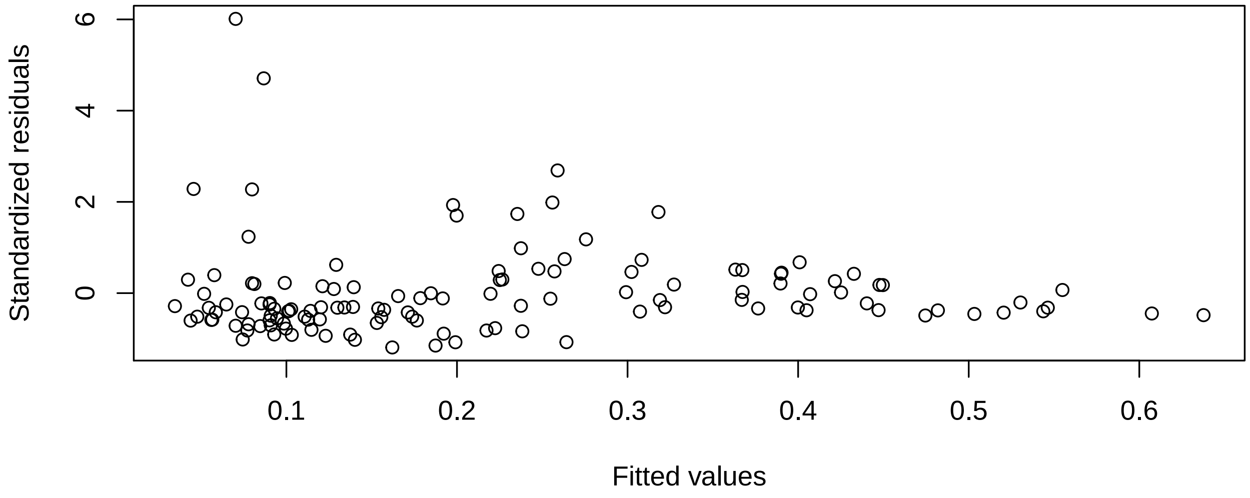

Figure 18: Residuals vs. fitted values for M-Index

That fixes the heteroescedasticity issue.

Corrected

We then apply a correction for ancestry and re-analyze the data.

Like with curvature and eccentricity, the relationship between curvature and skin pigmentation is no longer

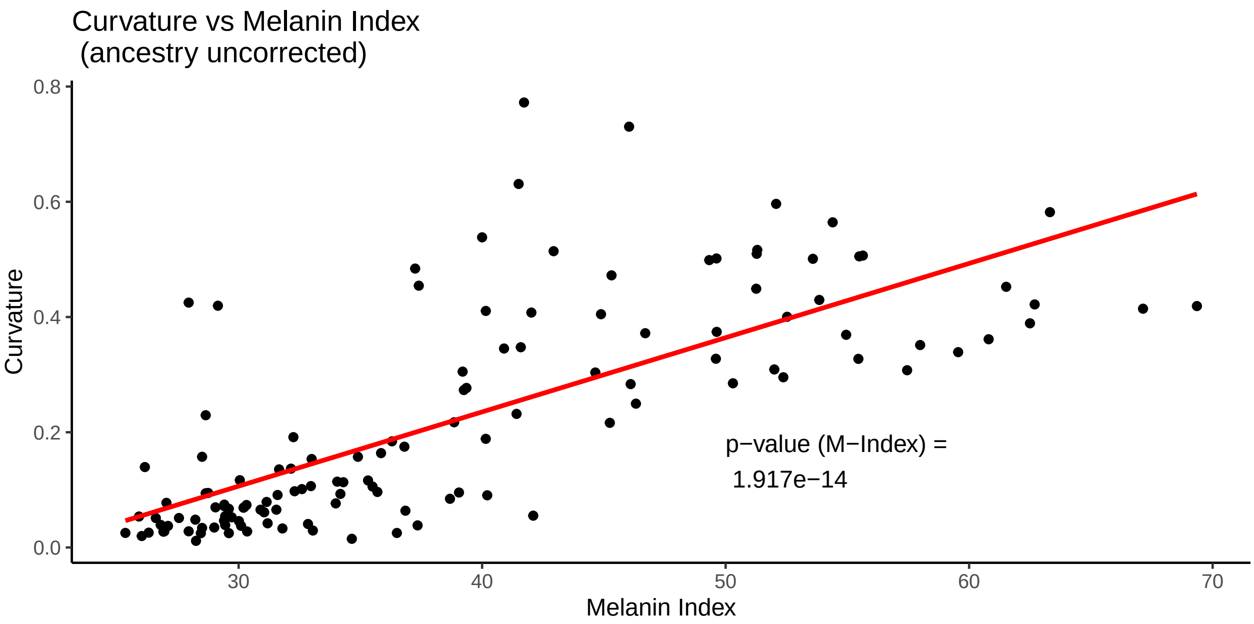

Figure 19: Curvature vs Melanin Index (ancestry uncorrected)

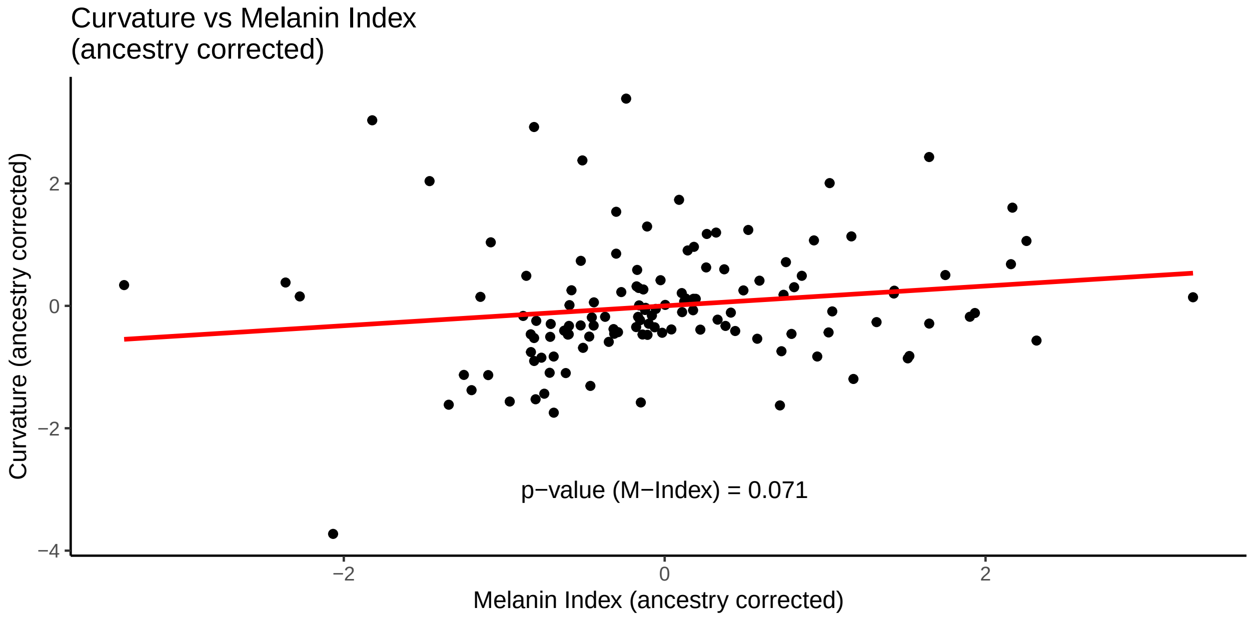

Figure 20: Curvature vs Melanin Index (ancestry corrected)

significant when ancestry is taken into account.
