# Supplementary material for: High-throughput phenotyping methods for quantifying hair fiber morphology
Source: Sci Rep. 2021 Jun 1;11:11535. doi: 10.1038/s41598-021-90409-x (PMC8169905; doi:10.1038/s41598-021-90409-x)
Supplement: Supplementary file 4 — Supplementary Information 4. [file 41598_2021_90409_MOESM4_ESM.pdf]

# High-throughput phenotyping methods for quantifying hair fiber morphology

**Tina Lasisi[1]*, Arslan A. Zaidi[2], Timothy H. Webster[3], Nicholas B. Stephens[1], Kendall Routch[1], Nina G. Jablonski[1], Mark D. Shriver[1]**

[1] Department of Anthropology, Pennsylvania State University
[2] Department of Genetics, Perelman School of Medicine, University of Pennsylvania
[3] Department of Anthropology, University of Utah
* Tina Lasisi: tpl5158@psu.edu

# Supplementary Videos

View and download from Box folder: https://psu.box.com/s/eb0qs1y5b8dhm5fprjht38qdsawhyt3a

**Supplementary Video 1.** Video of sample preparation methods for cross-sectional images of hair. Hair is embedded in a low melt-point plastic and sectioned in preparation for imaging with a microscope.

Link to YouTube: https://youtu.be/OSgiErHmiDE
Link to Box file: https://psu.box.com/s/paab2n16aj4vtjap0f1txcnkg32lfxni

**Supplementary Video 2**. Video of sample preparation for curvature images of hair. Hair is cut into fragments, washed and decanted into Petri dish with isopropanol for macroscopic imaging.

Link to YouTube: https://youtu.be/qR-Jj4K0Bts
Link to Box file: https://psu.box.com/s/6jgvk68wdg3uadwi1006f72fzep5ozz8
